# Tissue oedema following pulsed field ablation recognized during a concomitant left atrial appendage closure procedure: a case report

**DOI:** 10.1093/ehjcr/ytae495

**Published:** 2024-09-10

**Authors:** Gemma Gaggiotti, Stefano Bordignon, Shota Tohoku, Boris Schmidt, Julian Kyoung-Ryul Chun

**Affiliations:** Cardiology and Arrhythmology Clinic, University Hospital Ospedali Riuniti Umberto I-Lancisi-Salesi, Via Conca 71, 60126, Ancona, Italy; Cardioangiologisches Centrum Bethanien (CCB) Frankfurt am Main, Medizinische Klinik III, Agaplesion Markus Krankenhaus, Wilhelm-Epstein-Straße 4, 60431, Frankfurt am Main, Germany; Cardioangiologisches Centrum Bethanien (CCB) Frankfurt am Main, Medizinische Klinik III, Agaplesion Markus Krankenhaus, Wilhelm-Epstein-Straße 4, 60431, Frankfurt am Main, Germany; Cardioangiologisches Centrum Bethanien (CCB) Frankfurt am Main, Medizinische Klinik III, Agaplesion Markus Krankenhaus, Wilhelm-Epstein-Straße 4, 60431, Frankfurt am Main, Germany; Cardioangiologisches Centrum Bethanien (CCB) Frankfurt am Main, Medizinische Klinik III, Agaplesion Markus Krankenhaus, Wilhelm-Epstein-Straße 4, 60431, Frankfurt am Main, Germany; Cardioangiologisches Centrum Bethanien (CCB) Frankfurt am Main, Medizinische Klinik III, Agaplesion Markus Krankenhaus, Wilhelm-Epstein-Straße 4, 60431, Frankfurt am Main, Germany

**Keywords:** WATCHMAN FLX™ device (Boston Scientific, Plymouth, MN, USA), Pulsed field ablation (PFA), Case report, Left atrial appendage closure (LAAC)

## Abstract

**Background:**

In patients with non-valvular atrial fibrillation (AF), at high stroke risk, and who are ineligible for long-term oral anticoagulation, the left atrial appendage closure (LAAC) could be an alternative to anticoagulation. Pulsed field ablation (PFA) is a new non-thermal method for cardiac ablation modality based on high-voltage electrical energy for irreversible electroporation. We first report a case of a concomitant PFA pulmonary vein isolation (PVI) and LAAC.

**Case summary:**

A 74-year-old female patient was referred to our department for PVI for persistent AF (CHA_2_DS_2_-VASc score 5). A concomitant percutaneous LAAC was proposed because of a history of previous cerebellar transient ischaemic attack despite continuous oral anticoagulation therapy. Pulmonary vein isolation was achieved with a pentaspline PFA catheter, and LAAC was performed with a WATCHMAN FLX™ device (Boston Scientific, Plymouth, MN, USA). After PVI, a swelling of the left atrial ridge was observed, yet a 27 mm LAAC device was successfully implanted. The follow-up transesophageal echo (TEE) after 6 weeks showed complete resolution of the oedema, no device-related thrombus, but a slight proximal tilting of the LAAC device without leakage could be observed. The 6-month follow-up demonstrated a stable sinus rhythm, no stroke, or bleeding events were recorded.

**Discussion:**

In this case of synchronous PFA-PVI procedure in AF and WATCHMAN FLX™ device implantation, the electroporation created an acute oedema at the ridge level which at the TEE follow-up after 6 weeks was resolved. This resulted in a slightly tilted WATCHMAN device position which was nevertheless stable and showed no leakage.

**Learning points**

- Pulsed field ablation (PFA) is a novel non-thermal energy for pulmonary vein isolation.
- This is a case of concomitant PFA-PVI and LAAC in a patient with stroke despite continued oral anticoagulation. A swelling of the LAA-PV ridge was observed, not precluding the success of LAAC.

## Introduction

Pulsed field ablation (PFA) is a non-thermal ablation modality for the treatment of atrial fibrillation. This energy ablates specifically myocardium through irreversible electroporation sparing the oesophagus and/or phrenic nerves.^1–4^ In patients with non-valvular atrial fibrillation (AF), an estimated 90% of thrombi originate from the left atrial appendage (LAA).^5^ In the STR-OAC study, the left atrial appendage closure (LAAC) demonstrated to be an interventional therapeutic option for patients with stroke despite of oral anticoagulation.^6^ A combined procedure with percutaneous LAAC by a WATCHMAN device and pulmonary vein isolation (PVI) has been described in cryoballoon ablation^7^, and a prospective randomized control trial is underway (OPTION study).^8^ Herein, we discuss a case of concomitant PFA-PVI and LAAC procedure. A swelling of the LAA-PV ridge was observed, not precluding the success of LAAC.

## Summary figure

**Figure:**
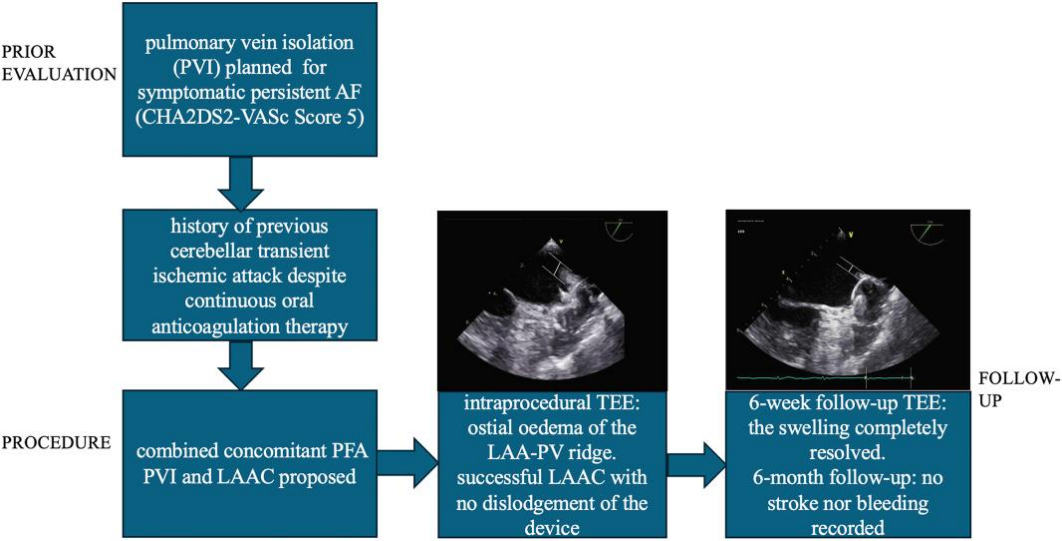


## Case presentation (clinical setting and method)

A 74-year-old female patient (body mass index 22 kg/m^2^), with arterial hypertension, previous ablations of an accessory pathway, and a slow AV node pathway, was referred to our department for PVI and symptomatic persistent AF. The patient also suffered a transient ischaemic attack with dysarthria (cerebellar) despite continuous oral anticoagulation with dabigatran (150 mg twice a day), so she had a CHA_2_DS_2_-VASc score of 5. To achieve rhythm control and to improve stroke prevention, a combined PVI and LAAC was proposed and accepted by the patient. A diagnostic multipolar catheter (6-F, Inquiry, Abbott, California) was positioned in the coronary sinus. A single transseptal puncture (SL1, 8.5-F, Abbott) was performed under fluoroscopy guidance. After pulmonary vein (PV) angiograms, a 31 mm PFA ablation catheter (Farawave®, Farapulse Inc., USA) was selected. The transseptal sheath was exchanged with the 13-F steerable delivery sheath (Faradrive®, Farapulse Inc., USA) over a wire in the left superior PV (LSPV). Each PV was ablated with a 10 applications protocol (4 in flower and 4 in basket configuration + 2 additional applications in olive shape). The ‘olive shape’ applications were performed orthogonally with the Farawave® catheter in a basket configuration with a reduced diameter, positioned relatively deeper at the PV ostium. This dosing protocol adding 2 further applications was adopted aiming for a higher PVI durability, since ‘real world data’ showed a lower rate of durable PV isolation compared with the initial reports.^9^

After the last ablation, sinus rhythm was restored with electrical cardioversion. The PV electrical isolation was confirmed with entrance block at the PV ostia performed by the Farawave® in an olive shape. A transesophageal echo (TEE) probe was therefore inserted to guide LAAC. Interestingly, a swelling of the ridge between LSPV and LAA was observed: we interpreted this swelling as an oedema that did not seem to involve the landing zone, so we proceeded with the LAAC procedure.

**Figure 1:**
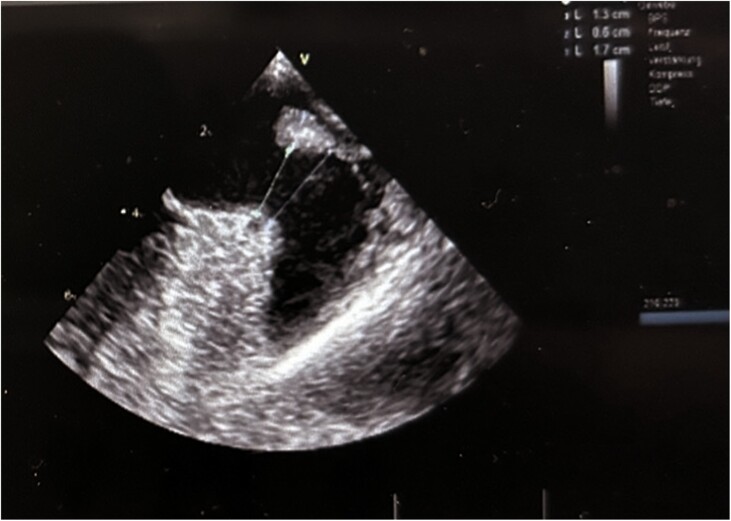
The maximal diameter of the left atrial appendage landing zone was 22.8 mm as measured on transesophageal echo.

**Figure 2:**
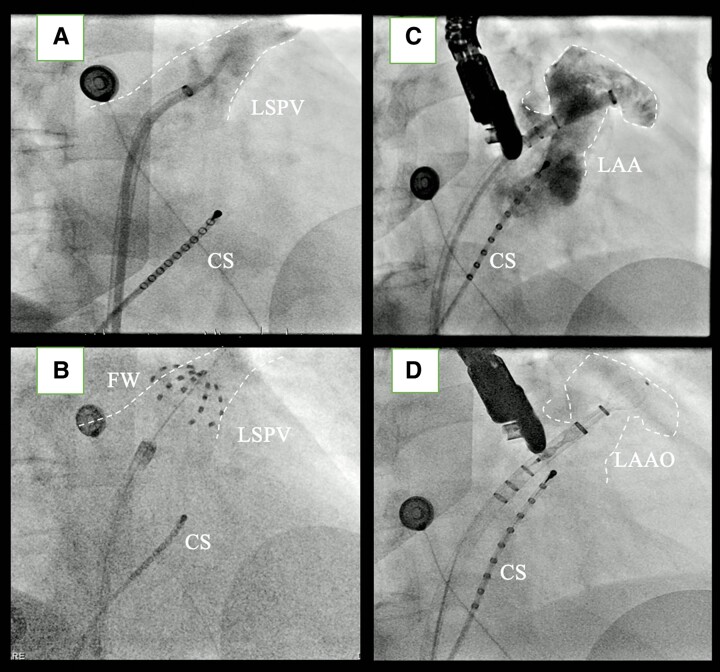
(*A*) Fluoroscopic image of the angiogram of left superior pulmonary vein in right anterior oblique for LSPV. (*B*) Fluoroscopic image of pulmonary vein isolation with the 31 mm pulsed field ablation catheter (Farawave®) in the left superior pulmonary vein in right anterior oblique 30°. (*C*) Fluoroscopic image of the angiogram of the left atrial appendage in right anterior oblique 30°-cranial 15°. (*D*) Fluoroscopic image of the left atrial appendage occlusion in right anterior oblique 30°-cranial 15° with the WATCHMAN FLX™ device 27 mm. Is visible the intraprocedural oedema of the LAA-PV ridge. In all the images is visible the diagnostic multipolar inquiry catheter positioned in the coronary sinus. LSPV, left superior pulmonary vein; PV, pulmonary vein; LAA, left atrial appendage; CS, coronary sinus; LAAO, left atrial appendage occlusion; FW, Farawave® catheter.

The Faradrive® sheath was exchanged for a Boston Scientific double curve 14 Fr delivery sheath over a wire in the LSPV. A 6 Fr pigtail catheter was utilized to direct the WATCHMAN 14 Fr delivery sheath in the LAA, and a selective angiography was performed in the right anterior oblique 30°, cranial 15°, and caudal 20° view. The maximal diameter of the LAA landing zone on either angiography or TEE was 22.8 mm (*Figure 1*), so a WATCHMAN FLX™ 27 mm device was chosen. The device was advanced in the LAA (*Figure 2*) and could be successfully implanted at the first attempt without the need for repositioning. In the Doppler sequences, no residual flow could be noticed. The patient was discharged on a double therapy with dabigatran 150 mg twice a day as direct oral anti-coagulant (DOAC) + aspirin, and a TEE control was scheduled 6 weeks after the procedure. Interestingly, the TEE showed a complete resolution of the ridge oedema, with a slight proximal movement of the device. The compression remained 22 mm, and the occlusion was satisfying, so the DOAC therapy (dabigatran 150 mg twice a day) was interrupted, and the aspirin continued. During the 6 months follow-up, no stroke nor bleeding was recorded and no recurrent AF was recorded.

## Discussion

Pulsed field ablation is an emerging non-thermal energy for cardiac ablation and demonstrated to be safe and effective.^9,10^ Percutaneous LAAC is an alternative therapeutic option in patients with AF with a thromboembolic event and/or LAA thrombus despite OAC treatment.^11^ This case proposes a combined intervention to reduce stroke risk following the findings of the STR-OAC trial.^6^

**Figure 3:**
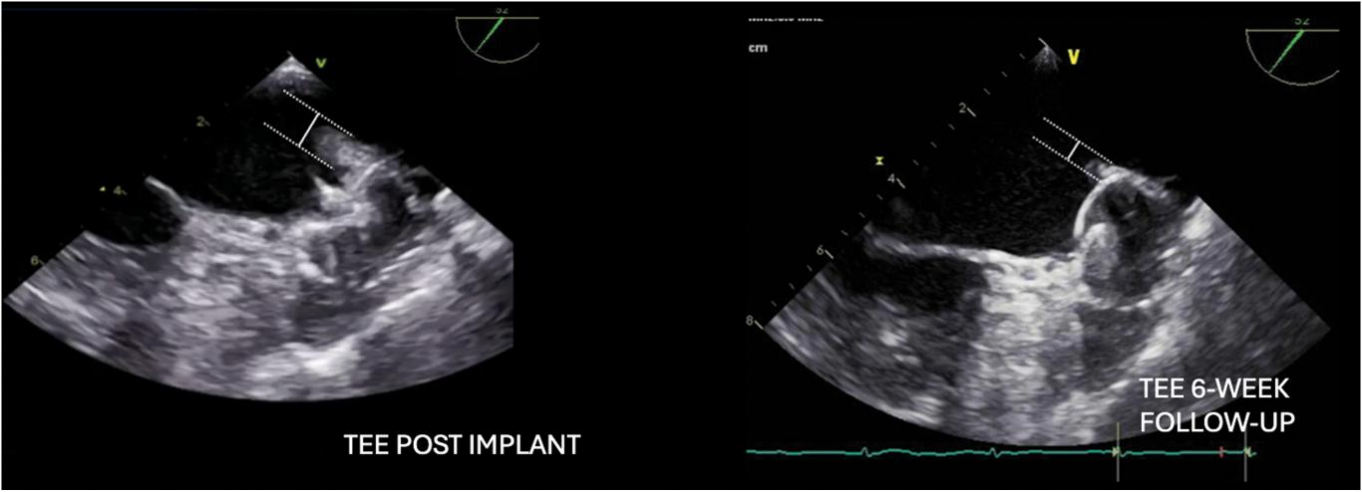
Transesophageal echo images of the marked oedema (0.8 cm) of the LAA-PV ridge observed during the procedure compared with the 6-week follow-up transesophageal echo images where the swelling completely resolved with an oedema of the measure of 0.4 cm. TEE, transesophageal echo.

Concomitant LAAC and PVI with thermal ablation was already described.^12^ Device implantation on tissue recently ablated may expose to the risk of dislodgment of the device with peri-device leaks. However, the Surpass Registry reported no difference in the extent of peri-device leaks at 45-day follow-up between combined PVI and LAAC and LAAC alone.^13^ Since PFA has a different mechanism for lesion formation,^9^ we expected to observe less oedema at the LAA basis that could interfere with LAA closure. As demonstrated in *Figure 3*, this was not the case: a marked oedema of the LAA-PV ridge (site of direct contact to tissue: basket/olive configuration) was observed (0.8 cm), particularly clear if compared with the 6-week follow-up TEE images where the swelling completely resolved (0.4 cm). The oedema deemed to be ostial and not within the landing zone, so the LAAC was accomplished uneventfully. However, careful comparison of the TEE follow-up images revealed a small proximal tilting, not impacting the sealing of the LAA nor the stability of the device.

It must be noticed that we opted for a Watchman FLX™ occlusion device, which can be implanted in a relative deeper landing zone compared with alternative device with a lobe/disk configuration. If such devices are used, a deeper implantation of the lobe should be suggested. In fact, since the oedema was interesting the proximal part of the ridge, a proximal device implantation may lead to missizing and dislocation. Therefore, one can speculate that a landing zone not interested by the oedema may increase the stability of the device in the follow-up. Before a concomitant procedure can be largely utilized, more data are needed. The results of the randomized OPTION trial (Comparison of Anticoagulation with Left Atrial Appendage Closure after AF Ablation)^8^ will help clarify the risk benefit of such a procedure. Nevertheless, in the OPTION study, PFA was not available. The mechanism underlying oedema after PFA is ill defined by the literature, but oedema following immediately PFA was described in pre-clinical studies: in porcine model, electroporation induced acute myocardial lesions with contraction band necrosis and interstitial oedema.^14^ The creation of nanopores alters the cellular permeability, a possible cause of oedema. Oedema following thermal ablation is a well-known phenomenon, likely due to increased capillary permeability produced by the heating energy.^15^ However, no haemorrhage, protein denaturation, and cardiomyocyte swelling as seen in the ablation with radiofrequency energy were described after PFA.^14^ The difference in oedema formation between thermal energies and PFA should be further studied.

In conclusion, our case shows the feasibility of concomitant PFA-PVI and LAAC with a WATCHMAN device. Nonetheless, even after a non-thermal energy, like PFA, a substantial ridge oedema can occur. The observation of the oedema should be considered in concomitant LAAC procedures, with regard to device size selection and implantation strategy. In the present case, this swelling did not result in peri-device leak or dislodgement, but larger series are needed.

## Data Availability

The data underlying this article will be shared on reasonable request to the corresponding author.

## Lead author biography

Dr Gemma Gaggiotti is a cardiology resident at Cardiology and Arrhythmology Clinic, University Hospital Ospedali Riuniti Umberto I-Lancisi-Salesi, Ancona, Italy. Her main areas of interest are interventional cardiology and structural heart disease.

## Notes

**Consent:** Informed consent was obtained from the patient for the publication of this case report in line with the COPE guidelines.

**Funding:** None declared.
